# The dynamic interaction of pediatric ALL cells and MSCs: influencing leukemic cell survival and modulating MSC β-catenin expression

**DOI:** 10.1007/s00418-025-02353-w

**Published:** 2025-01-21

**Authors:** Tuba Ozdemir-Sanci, Ilkay Piskin, Yasin Köksal, Sevil Cayli, Namik Y. Ozbek, H. Meltem Ozguner

**Affiliations:** 1 Department of Histology and Embryology, Faculty of Medicine, Ankara Yildirim Beyazit University, 06800 Ankara, Turkey; 2 Stem Cell Research Laboratory, Ankara City Children’s Hospital, Ankara, Turkey; 3 Department of Pediatric Hematology and Oncology, Ankara City Children’s Hospital, Ankara, Turkey

**Keywords:** Acute lymphoblastic leukemia, Bone marrow microenvironment, Bone marrow mesenchymal stromal cells, Co-culture, Leukemic blast, β-catenin

## Abstract

Bone marrow mesenchymal stromal cells (BM-MSCs) are integral components of the bone marrow microenvironment, playing a crucial role in supporting hematopoiesis. Recent studies have investigated the potential involvement of BM-MSCs in the pathophysiology of acute lymphoblastic leukemia (ALL). However, the exact contribution of BM-MSCs to leukemia progression remains unclear because of conflicting findings and limited characterization. In this study, we compared BM-MSCs derived from pediatric ALL patients with those from matched healthy donors (HDs). Our results indicate that while both ALL-MSCs and HD-MSCs meet the criteria established by the International Society for Cellular Therapy, they exhibit significant differences in proliferation and differentiation capacity. ALL-MSCs displayed markedly lower proliferation rates and reduced osteogenic/adipogenic differentiation potential compared to HD-MSCs. Furthermore, co-culture experiments revealed that MSCs enhance the survival of leukemic blasts through both soluble factors and direct cell-cell interactions, underscoring their anti-apoptotic properties. Importantly, our findings demonstrate that interactions with leukemic cells activate the Wnt/β-catenin signaling pathway in MSCs, suggesting a potential target for therapeutic intervention. Overall, this study enhances our understanding of the role of BM-MSCs in leukemia and highlights β-catenin as a promising target for future therapies.

## Introduction

Leukemic blasts, which are malignant B and T cells originating in the bone marrow, are the primary drivers of acute lymphoblastic leukemia (ALL), a hematologic disease most commonly found in children. Despite significant advances in the understanding of acute leukemia and the development of novel treatments, a substantial number of ALL patients experience relapse or develop resistance to treatment, resulting in poor outcomes (Yang et al. 2013).

Mesenchymal stromal cells (MSCs), located in the bone marrow stroma alongside hematopoietic cells, play a pivotal role in the leukemia progression by promoting the growth and persistence of leukemic blasts (Lim et al. 2019). Through direct interaction with hematopoietic cells or the secretion of various growth factors and cytokines, MSCs regulate the survival and expansion of leukemic blasts. Many studies have focused on leukemic cell survival based on these interactions within the bone marrow microenvironment (Ayala et al., 2009; Conforti et al. 2013). However, the dual roles of MSCs in both promoting and suppressing tumor growth complicate their adaptation in anticancer therapies. Minimal information is available comparing the antitumorigenic and protumorigenic activities of MSCs in hematologic malignancies compared to solid tumors (Wong and Cheong 2014; Lee et al. 2019).

Beyond their biologic abnormalities, MSCs play two key roles in leukemogenesis: leukemic cells can either disrupt the bone marrow microenvironment or MSCs can protect leukemic cells, accelerating their growth. This bidirectional interaction between leukemic cells and MSCs may cause MSCs to transform into malignant forms (Wong and Cheong, 2014). Additionally, the interaction between MSCs and leukemic blasts may influence molecular pathways regulating hematopoiesis.

The Wnt signaling pathway is critical to many biologic processes, including embryonic development, hematopoietic stem cell proliferation, cell differentiation, and tissue formation (Pehlivan et al. 2018). The canonical Wnt pathway, mediated primarily by β-catenin, plays a central role in these processes (Hatırnaz Ng et al. 2015). β-Catenin is essential for the structural integrity and functional polarization of epithelial and other tissues (Buechel et al. 2021). Upon activation, β-catenin accumulates in the cytoplasm, translocates to the nucleus, and binds to oncogenic transcription factors (Ng et al. 2014). Previous studies have identified abnormalities in Wnt signaling as being associated with hematologic cancers, with changes in β-catenin expression observed in leukemic blasts following their interaction with MSCs in ALL, AML, CLL, and CML (Soares-Lima et al. 2020; Chiarini et al. 2020). However, the regulation of this pathway by MSCs, particularly how MSCs influence β-catenin expression under different culture conditions, remains poorly understood.

Understanding the interaction between MSCs and leukemic blasts in ALL could offer new therapeutic avenues by targeting cancerous cells through modification of the bone marrow microenvironment. Disruption of this microenvironment leads to several problems, including the evasion of therapeutic agents by leukemic cells (Zhang et al. 2021). Therefore, this study examines the impact of MSCs on the survival of leukemic blasts using MSCs derived from pediatric ALL patients (ALL-MSCs) and healthy donors (HD-MSCs). We co-cultured HD-MSCs and ALL-MSCs with B and T blasts under both contact and non-contact (Transwell) conditions. We hypothesized that direct contact with BM-MSCs would promote blast survival and alter Wnt signaling pathway activation. Specifically, we evaluated β-catenin expression to determine how leukemic blasts affect healthy and leukemic MSCs. Our findings demonstrate that MSCs exhibit anti-apoptotic effects on blasts and that leukemic blasts selectively activate the Wnt pathway in HD-MSCs, which may contribute to blast survival and persistence.

## Materials and methods

### Patient and healthy donor samples

Twelve healthy donors who were matched with 12 newly diagnosed pediatric ALL patients (9 pre-B-ALL, 3 T-ALL) treated at the Ankara City Children's Hospital. ALL patients with relapse and conditions affecting the bone marrow were excluded. Patients with relapsed ALL or conditions affecting the bone marrow were excluded from the study. Both patients and donors provided informed consent, and the study was approved by the local ethics committee (approval number: 2014-063). The ALL-BFM 2000 protocol was followed for the diagnosis, classification, and treatment of patients. The proportions of leukemic blasts were assessed microscopically using May-Grünwald Giemsa staining and by flow cytometry.

### Isolation of mononuclear cells from bone marrow and peripheral bood

After plasma collection, bone marrow samples were diluted with PBS (BiochromGmbH, Merck Millipore, Germany) and layered onto Biocoll™ separating solution (1.077 g/ml, BiochromGmbH, Merck Millipore, Germany) in a 1:1 ratio. Centrifugation was performed at 2000 rpm for 20 min to isolate the mononuclear cells (MNCs). The buffy coat, containing MNCs, was collected and subjected to a second centrifugation step. MNCs (25 × 10^6^) were seeded into 75-cm^2^ culture flasks following the protocol described in a previous study (Ok Bozkaya et al. 2015). Flasks were incubated at 37 °C in a humidified atmosphere with 5% CO₂, and the culture medium was refreshed every 3 days. The same procedure was used to isolate MNCs from peripheral blood. T and B leukemic cells were isolated from peripheral blood using the EasySep™ Human B and T Cell Isolation Kit (STEMCELL Technologies, Canada Inc.) for immunomagnetic negative selection. After isolation, cells were counted using a Beckman Coulter HmX-AL cell counter, and their characteristics were analyzed by flow cytometry.

### MSCs harvest and culture

Mononuclear cells were cultured using DMEM-LG (Dulbecco's Modified Eagle Medium–Low glucose) supplemented with 10% fetal bovine serum (BiochromGmbH, Merck Millipore, Germany) and 1% penicillin/streptomycin (BiochromGmbH, Merck Millipore, Germany) for 96 h, with non-adherent cells being removed after 3 days. Adherent cells were allowed to proliferate, and once they reached 90% confluence, they were detached using a 0.25% trypsin/EDTA solution 0.02% in PBS, without Ca2 + , Mg2 + (Biochrom GmbH, Merck Millipore, Germany). The cells were passaged until the second passage (P2). During this passage, MSC differentiation tests and characterization assays were conducted.

### Cell viability and proliferation capacity of MSCs

Each passage of MSCs was counted and reseeded at a density of 5 × 10^3^ cells per cm^2^. Cell proliferation was monitored until passage 5. The number of population doublings (PDs) was calculated using the formula log10(N)/log10(2), where "N" is the ratio of cells harvested to cells seeded. Cell viability at each passage was assessed using Trypan Blue (Invitrogen) exclusion.

### Characterization and Differentiation Assay of MSCs

MSCs were cultured in T25 flasks for 21 days to induce osteogenic and adipogenic differentiation. Once cells reached 100% confluence, they were washed with PBS, and MesenCult® Adipogenic and Osteogenic Stimulatory Supplements (STEMCELL Technologies, Canada) were added. After 3 weeks, cells were fixed in formalin and stained with Oil Red O (Sigma, USA) and Alizarin Red (Sigma-Aldrich) to assess adipogenic and osteogenic differentiation, respectively. Images were captured using an Olympus IX73 microscope with Olympus cellSens imaging software at 10 × magnification (NA:0.25/W.D:10 mm). The presence of adipocytes was determined using Oil Red O staining, an indicator of intracellular lipid accumulation. Lipid droplets were counted in five different fields, and the percentage of Oil Red O-positive MSCs was calculated by averaging the percentages. The analysis of osteogenic differentiation was performed by counting the bone nodules observed in five different fields under a light microscope (Olympus IX73). The counting was conducted independently by two different researchers to ensure accuracy and eliminate observer bias (Azadniv et al. 2020).

MSC surface markers were also analyzed using flow cytometry. Cells were stained with CD45-Alexa Flour® 488 (BioLegend, cat. no. 304019), CD34-FITC (BioLegend, cat. no. 343604) antibody staining and positive CD90-PE (BioLegend, cat. no. 328110), CD73-APC (BioLegend, cat. no.344006), and CD105-PE/Cy7 (BioLegend, cat. no. 323218) and analyzed on Navios-Beckman Coulter flow cytometer using Navios Software v1.2.

### Co-culture experiments

ALL blasts matched with their corresponding MSCs from the same patient in co-culture experiments. Leukemic blasts isolated from patients using EasySep™ B and T Cell Isolation Kit were co-cultured with passage 2 MSCs in 12-well plates (Cellstar, Greiner Bio-One). Two approaches were used: (1) direct co-culture: 2.5 × 104 MSCs were cultured, and after 24 h, 2.5 × 10^4^ blasts were added; (2) indirect co-culture: MSCs were seeded on the lower side of a Transwell system with 0.8-µm pore size (ThinCertTM Greiner Bio-One, Austria), and blasts were seeded on the upper side after 24 h. Following 96 h of co-culture, leukemic blasts were collected for flow cytometry analysis of apoptosis.

### Annexin V/PI apoptosis assay

After 4 days of co-culture, leukemic blasts were stained with Annexin V-FITC and propidium iodide (BioLegend, cat. no. 640914) following the manufacturer’s protocol. The leukemic blasts were briefly rinsed in 500 µl Annexin binding buffer, and then 7 µl propidium iodide and 5 µl annexin-V-FITC were added to the cell suspension. The cells were incubated for 20 min and analyzed in flow cytometry (Navios Beckmann Coulter).

### Immunocytochemistry

After co-culture, leukemic blasts were removed, and MSCs were fixed with 4% paraformaldehyde (Sigma-Aldrich) for 10 min at room temperature. After the blocking step, cells were incubated overnight with primary antibody β-catenin (1:50, E247, Abcam). The next day, cells were stained with an HRP-conjugated anti-rabbit IgG secondary antibody (1:500, Abcam). Cells were visualized using DAB staining (3,3'-diaminobenzidine) (Abcam) and imaged with an Olympus BX43 microscope equipped with an Olympus DP21 digital camera using a 40 × objective (NA: 0.65/W.D: 0.6 mm) and 20 × objective (NA: 0.4/WD: 1.2 mm).

### Statistical analysis

Immunohistochemical labeling was evaluated using the Image J IHC Profiler program. β-Catenin staining intensity was scored as follows: 0 (no staining), 1 + (weak staining), 2 + (moderate staining), and 3 + (strong staining). Five randomly selected slides from each group were analyzed. Statistical differences between groups were assessed using the Student’s *t*-test or one-way ANOVA. A *p*-value < 0.05 was considered statistically significant.

## Results

### Demographic and clinical characteristics of the study group

The median age of the ALL group was 8 (4–16) years, while the healthy donor group's median age was 14 (3–18) years (Table 1). The healthy donor group consisted of four girls and eight boys, whereas the ALL group included five girls and seven boys. There were no significant differences in age or sex between the two groups (Table 1).

**Table 1:** Demographic and hematologic data for patients and donors, including values collected at the time of diagnosis

Parameter	Patient	Donor
Age (mean ± SD)	9.6 ± 4.5	12.3 ± 5.8
Gender (*n*)		
Female	4	5
Male	8	7
Differential blood count (mean ± SD)		
Hemoglobin	8.8 ± 3.2	14.5 ± 1.5
WBC (1/mm^3^)	28,030 ± 33,956	7670 ± 2540
Blast count	20,809 ± 28,252	–
BM blast percentage	90.7 ± 9.3	–
Chromosomal abnormalities		
Favorable	1	
No abnormality	11	
Subtypes of ALL		
B cell lineage	9	
T cell lineage	3	
Remission status after induction therapy		
Remission	10	
No remission	2	

### Proliferation and differentiation capacity of MSCs: ALL vs. healthy donors

**Fig. 1:**
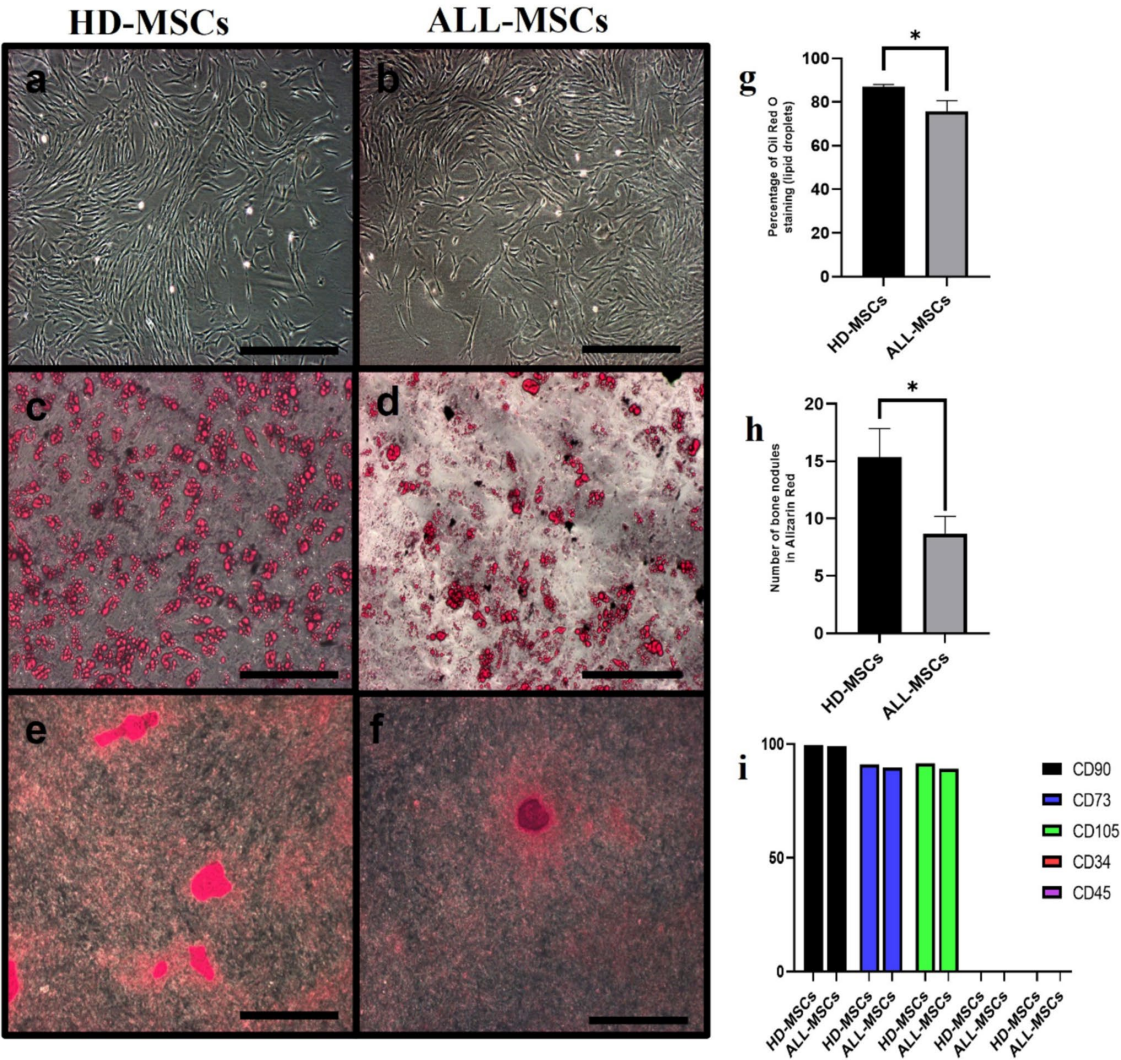
The biologic characteristics of ALL- and HD-MSCs at passage 2. **a** HD-MSC morphology; **b** ALL-MSC morphology (× 10, scale bar 100 µm). MSCs obtained from a single representative HD and a single ALL patient at diagnosis for their ability to differentiate into osteogenic and adipogenic tissues.**c** Development of adipocytes in HD-MSCs; **d** development of adipocytes in ALL-MSCs; **e** differentiation into osteoblasts in HD-MSCs; **f** differentiation into osteoblasts in ALL-MSCs (× 10). **g** Oil Red O staining % histograms in HD- and ALL-MSCs; **h** bone nodule histograms in HD- and ALL-MSCs; **i** surface marker expression histograms for both ALL- and HD-MSCs. *p < 0.05

**Fig. 2:**
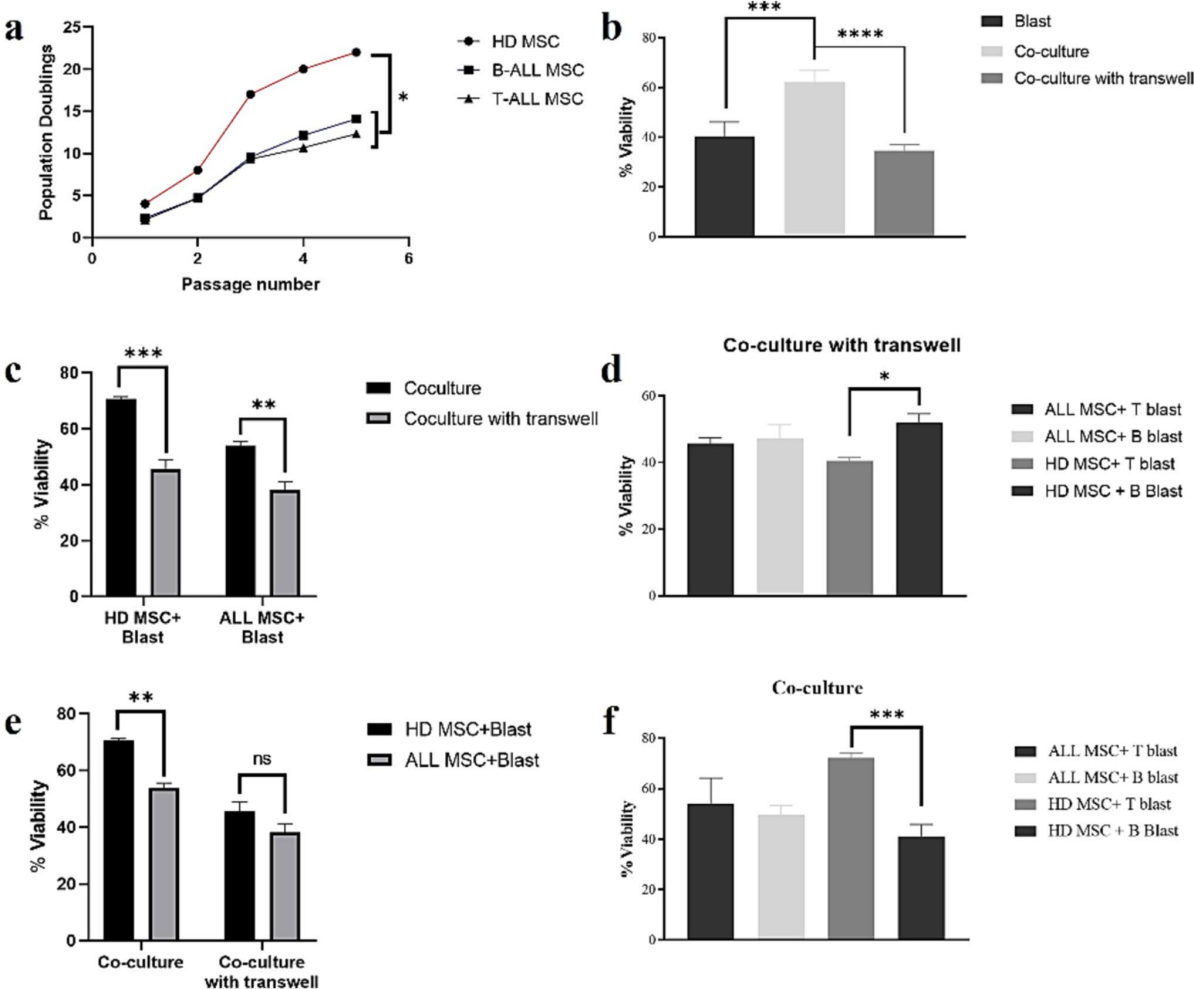
Proliferative capacity of MSC and ALL cell viability. **a** Population doublings of MSCs. **b** Viability of leukemic blasts in three different culture conditions. **c** Viability of blasts cultured with ALL- and HD-MSCs in co-culture conditions. **d** Viability of blasts cultured with ALL- and HD-MSCs in co-culture Transwell conditions. **e** Viability of leukemic B and T blasts cultured with ALL-MSCs and HD-MSCs in direct co-culture conditions. **f** Viability of leukemic B and T cells cultured with ALL-MSCs and HD-MSCs in co-culture Transwell conditions. ****p < 0.0001, ***p < 0.001, **p < 0.01, and *p < 0.05, ns: non-significant

Mesenchymal stromal cells were sourced from 12 ALL patients upon initial diagnosis to mitigate the effects of chemotherapy. Additionally, MSCs from 12 healthy donors were isolated for comparison. ALL-MSCs exhibited a similar elongated, fibroblast-like morphology and adhered to plastic surfaces, with no noticeable differences between ALL-MSCs and HD-MSCs (Fig. 1a, b). When assessing their expansion potential under identical in vitro culture conditions, it was evident that HD-MSCs had a significantly greater capacity for proliferation compared to ALL-MSCs (Fig. 2a).

We assessed the MSCs' potential to differentiate into osteocytes and adipocytes using specific culture media. Adipogenic differentiation was verified by Oil Red staining, which detected the existence of lipid vesicles in adipocyte cytoplasm (Fig. 1c, d). Alizarin Red staining revealed the creation of a mineralized matrix, indicating that MSCs underwent osteogenic differentiation (Fig. 1e, f). Comparisons of the adipogenic differentiation capabilities of MSCs from healthy donors (87%) and ALL patients (72.3%) during the same timeframe demonstrated that HD-MSCs exhibited a higher capacity for differentiation (*p* = 0.0175) (Fig. 1g). Similarly, quantifying bone nodules revealed a higher differentiation rate for MSCs obtained from healthy donors compared to ALL-MSCs (*p* = 0.0172) (Fig. 1h).

### Immunophenotypic comparison of ALL- and HD-MSCs

MSCs were isolated and expanded as previously outlined. At passage 2 (P2), their characteristics were assessed according to the minimal criteria for MSCs derived from bone marrow. The immune profile of MSCs was analyzed using flow cytometry, revealing that both ALL-MSCs and HD-MSCs expressed typical MSC markers while lacking hematopoietic markers (Fig. 1i). Although there were variations in the levels of CD105 and CD73 expression among ALL-MSCs, these differences did not reach statistical significance. Therefore, our findings indicate that ALL-MSCs exhibit similar immunophenotypes to HD-MSCs.

### MSC-mediated leukemic cell survival in co-culture models

To investigate the effect of MSCs on leukemic cell survival, blasts from 12 ALL patients were co-cultured with either ALL-MSCs or HD-MSCs. A Transwell insert system was used to create a non-contact culture environment, allowing us to distinguish between the effects of soluble factors and direct cell-to-cell contact.

After 4 days of co-culture, we assessed the viability of leukemic blasts using Annexin V/PI staining. In the absence of MSCs, leukemic cells underwent apoptosis when cultured in medium alone (Fig. 2). However, when co-cultured with MSCs, their survival increased significantly (40.4% ± 19.2% of leukemic cells in medium alone vs. 62.2% ± 25.2% in the presence of MSCs, *p* < 0.0001), highlighting the protective role of MSCs. Direct co-culture with MSCs resulted in higher survival rates compared to non-contact conditions using a Transwell system (62.2% ± 25.2% in direct co-culture vs. 34.6% ± 13.4% in the Transwell system, *p* < 0.001), indicating that cell-cell contact is crucial for promoting leukemic cell survival (Fig. 2b).

The viability of blasts was significantly higher in direct co-culture conditions compared to co-culture with Transwell for both HD MSCs and ALL MSCs (70.7% ± 1.8% and 45.7% ± 2.5%, respectively). Specifically, direct co-culture with HD MSCs resulted in a greater increase in blast viability compared to ALL MSCs. This difference was statistically significant (*p* = 0.0008 for HD MSC + Blast and *p* = 0.0047 for ALL MSC + Blast), suggesting that direct cell-to-cell contact plays a crucial role in promoting blast survival (Fig. 2c). Furthermore, the overall higher viability observed with HD MSCs compared to ALL MSCs indicates that healthy MSCs provide a more supportive microenvironment for blasts, potentially through stronger physical interactions or enhanced local signaling mechanisms. Similarly, the viability of blasts in direct co-culture with HD MSCs was significantly higher than that observed with ALL MSCs (Fig. 2e, *p* = 0.0038). However, in the Transwell condition, there was no significant difference in blast viability between HD MSCs and ALL MSCs, indicating that the supportive effects of HD MSCs over ALL MSCs are primarily driven by direct cell-to-cell interactions rather than soluble factors alone. Together, these findings highlight the critical role of MSC-mediated physical contact in regulating blast survival and suggest that MSC functionality is compromised in ALL patients. Interestingly, T blasts showed higher viability when cultured with HD-MSCs compared to B blasts (72.2% ± 2.5% for T blasts vs. 41.2% ± 14.40% for B blasts, *p* < 0.001) (Fig. 2f). These findings suggest that T cells may benefit more from MSC-mediated protection than B cells, particularly in direct co-culture conditions.

### Wnt/β-catenin pathway activation in MSCs co-cultured with leukemic blasts

**Fig. 3:**
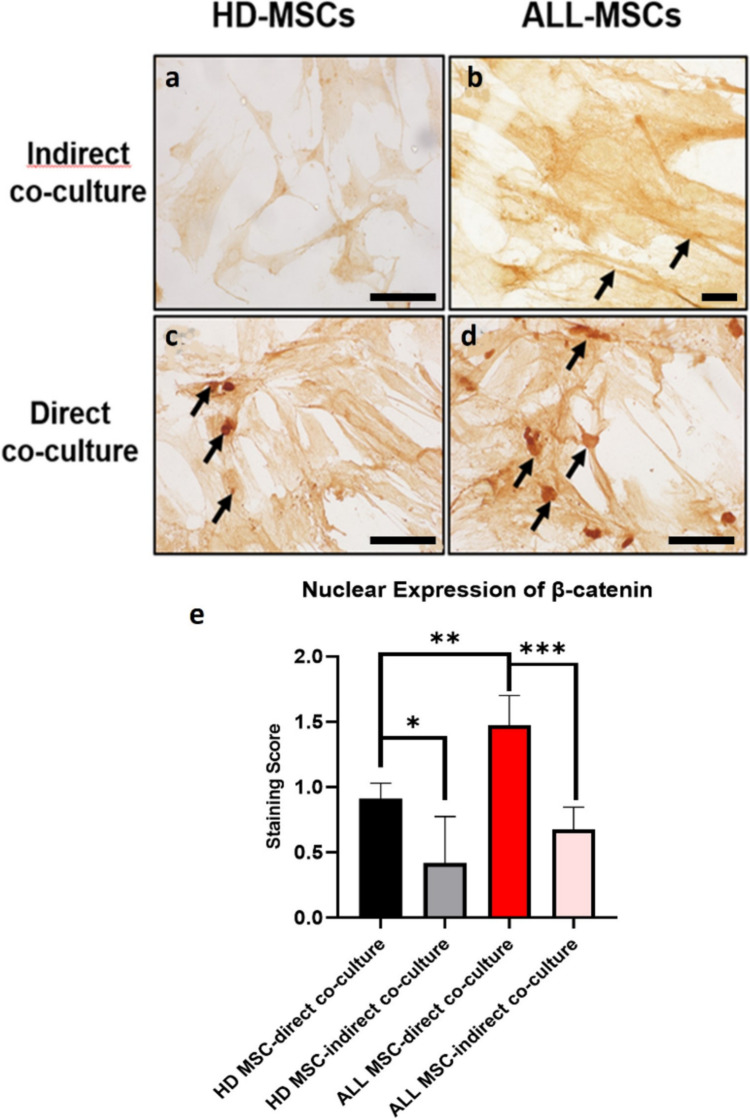
Activation of the Wnt/β-catenin signaling pathway in ALL- and HD-MSCs cultured with leukemic blasts. **a** β-Catenin staining in HD-MSCs (× 10, scale bar 100 µm). **b** β-Catenin staining in ALL-MSCs. **c** Nuclear β-catenin staining in ALL-MSCs (× 40, scale bar 25 µm). **d** Quantification of the percentage of β-catenin nuclear positivity in ALL-MSCs and HD-MSCs. **e** Quantification of β-catenin nuclear positivity in ALL-MSCs and HD-MSCs in direct and indirect co-culture conditions. The data are presented as mean ± SEM (****p < 0.0001, ***p < 0.001, and *p < 0.05)

We explored the activation of the Wnt/β-catenin signaling pathway in MSCs co-cultured with leukemic blasts by assessing the expression and localization of β-catenin (Fig. 3). Under normal physiologic conditions, β-catenin is located at the plasma membrane or within the cytoplasm. When β-catenin accumulates in the cytoplasm and translocates to the nucleus, it can activate oncogenic gene expression. In our study, MSCs under indirect co-culture conditions displayed weak to moderate cytoplasmic β-catenin immunoreactivity, with minimal nuclear staining. However, in direct co-culture, ALL-MSCs exhibited significantly higher nuclear β-catenin positivity compared to HD-MSCs (*p* = 0.037) (Fig. 3d). HD-MSCs exhibited higher expression levels in direct co-culture conditions compared to indirect co-culture conditions (*p* = 0.01) (Fig. 3e). Furthermore, in ALL-MSCs, nuclear β-catenin positivity increased significantly under direct co-culture conditions compared to indirect co-culture (*p* = 0.001), indicating that direct cell-cell interaction activates Wnt/β-catenin signaling in ALL-MSCs.

## Discussion

Bone marrow mesenchymal stromal cells (BM-MSCs) play a critical role in the bone marrow microenvironment, supporting hematopoiesis (Xia et al. 2020). Recently, there has been considerable interest in understanding the involvement of BM-MSCs in the pathophysiology of acute lymphoblastic leukemia (ALL). However, the exact role of BM-MSCs in supporting leukemia progression remains unclear because of conflicting research findings and limited characterization. In particular, it remains unclear how MSCs precisely contribute to the progression of leukemia.

In this study, we observed several differences between MSCs isolated from leukemia patients and those from healthy donors, consistent with previous research. These differences included variations in cell morphology (Geyh et al. 2016), growth rates (Conforti et al. 2013), differentiation capacity (Vicente López et al. 2014), and their ability to support hematopoietic stem and progenitor cells (Sorokina et al. 2016). We compared BM-MSCs derived from pediatric ALL patients with those from healthy donors (HDs), and while both groups met the minimal criteria proposed by the International Society for Cellular Therapy (Dominici et al. 2006), significant differences were noted in their proliferation and differentation capacities. These findings are consistent with those reported by Conforti et al., who described similar proliferative behaviors in MSCs derived from pediatric ALL patients and healthy donors (Conforti et al. 2013). We observed that one HD-MSCs showed a lower proliferative rate compared to others, and the proliferation rate of three ALL-MSCs was lower than the other ALL-MSCs. The variability in proliferation rates among MSCs, whether from healthy donors or leukemia patients, may be attributed to several factors, including the microenvironment, patient-specific characteristics, genetic background, and prior environmental exposures. In terms of differentiation, HD-MSCs exhibited greater osteogenic and adipogenic potential compared to ALL-MSCs. These results align with findings from Cheng et al. (2018), who demonstrated reduced ossification in leukemic mice. Conversely, Vanegas et al. (2021) reported only a modest reduction in osteogenic differentiation in MSCs associated with B-ALL. Interestingly, our study found no significant differences in the expression of surface markers between ALL-MSCs and HD-MSCs, corroborating the results of previous studies (Conforti et al. 2013; Vanegas et al. 2021). Notably, some studies have employed ALL cell lines instead of primary leukemic cells in their experiments (Garrido et al. 2001). By evaluating 12 patients devoid of chromosomal aberrations and contrasting their profiles with matched healthy donors, our study addresses existing gaps in the literature (Brenner et al. 2017). Previous research has shown that MSCs promote leukemic cell survival and inhibit apoptosis (Corradi et al., 2018), but the specific mechanisms involved remain unclear. We sought to determine whether MSCs contribute to leukemia initiation or whether leukemic blasts disrupt the microenvironment to promote disease progression. We aimed to determine whether the microenvironment induces the formation of leukemia or whether immature blasts disrupt the microenvironment. Studies indicate that the mesenchymal stem cells affect the survival of leukemic cells and even leukemic stem cells in acute myeloid leukemia (AML), chronic myeloid leukemia (CML), chronic lymphocytic leukemia (CLL), and ALL (Tesfai et al. 2012; Zuo et al. 2021; Patterson and Copland 2023). However, information on the effects of leukemic blasts in both the healthy microenvironment and the leukemic microenvironment is controversial. Our study found that leukemic blast viability was significantly higher in direct co-culture groups. This indicates that mesenchymal stromal cells possess anti-apoptotic properties not only through cytokine secretion but also through direct cell-cell interactions that enhance leukemic blast survival. The question of whether the normal microenvironment is compromised and contributes to the initiation and development of leukemia or leukemic cells that evade apoptosis adversely affect the healthy microenvironment remains unresolved (Dander et al. 2021). To address this question, we assessed leukemic cell viability by adding leukemic blasts to both healthy MSCs and leukemic MSCs. The results indicated that there is statistically significant difference in viability between the contact and non-contact groups for both healthy and leukemic cells. Our findings indicate that leukemic blast viability was significantly higher in direct co-culture conditions, suggesting that MSCs exert anti-apoptotic effects through both cytokine secretion and direct cell-cell interactions. This observation aligns with the hypothesis that direct interactions between leukemic cells and MSCs are critical for leukemic blast survival. Furthermore, direct co-culture with HD MSCs supports leukemic blast viability significantly better than co-culture with ALL MSCs (*p* < 0.01), while no significant differences were observed under Transwell conditions. These results suggest that the supportive role of MSCs in leukemic cell survival is compromised in ALL-derived MSCs, potentially because of intrinsic defects or alterations in their ability to provide a conducive microenvironment. Interestingly, when comparing T and B blasts, we found higher viability in T blast co-culture groups, likely because of the crucial role MSCs play in supporting T cell survival (Jimenez-Puerta et al. 2020).

Altered β-catenin expression has been extensively studied in leukemic blasts (Khan et al. 2007; Ng et al. 2014; Soares-Lima et al. 2020; Tan et al. 2022), with research indicating that its activation plays a crucial role in maintaining drug resistance in both AML and ALL (Chiarini et al. 2020). Typically, most β-catenin resides on the cytoplasmic side of the membrane, participating in cadherin-based cell-cell adhesions in the absence of Wnt stimulation (Yu et al. 2022). To activate the Wnt pathway, in ALL cells, MSCs must express Wnt ligands. Previous studies have explored the effects of MSCs on primary human ALL cells (Kamga et al. 2020; Ruan et al. 2020; Takam Kamga et al. 2021; Yu et al. 2022), but none focused on differences in nuclear β-catenin expression between leukemic and healthy MSCs after contact and non-contact culture conditions. Consequently, it remains unclear how leukemic cells modulate β-catenin expression in MSCs following interactions with healthy or leukemic MSCs. In our study, when assessing the co-culture of HD-MSC and ALL-MSCs, particularly in the direct co-culture groups, we observed notably high nuclear beta-catenin expression, irrespective of whether the MSCs originated from healthy donors or leukemia patients. Typically, in healthy MSCs, nuclear beta-catenin expression is minimal. However, the entry of leukemic cells into the environment leads to an increase in expression, suggesting that changes in the microenvironment significantly affect MSCs characteristics (Kim et al. 2015). The variation in β-catenin levels accumulating in MSCs distinctly regulates this behavior, leading to the differences in their ability to stimulate hematopoietic stem cells (HSCs) (Jeong et al. 2020; Ruan et al. 2020). Sun et al. conducted a study to investigate the role of β-catenin in leukemic cell survival by co-culturing three different ALL cell lines with MSCs, where they found that MSCs activated the Wnt pathway in ALL cells (Yang et al. 2013). Our findings contribute to these studies by being the first to our knowledge to demonstrate that the activation of the Wnt signaling pathway in pediatric ALL-MSCs and healthy MSCs in co-culture conditions may play a role in influencing leukemic cell survival (Takam Kamga et al. 2021).

Limitations of this study are the relatively small sample size and variability in patient demographics, such as age and ALL subtype. Furthermore, the in vitro co-culture models on which our analysis is based may not accurately represent the complexity of bone marrow in vivo and may have an impact on cell interactions mediated by extracellular matrix components and other cell types. Another limitation is that the study’s primary focus on the Wnt/β-catenin pathway does not encompass the investigation of other key proteins involved in this signaling pathway that may contribute to leukemic cell survival and resistance within the bone marrow niche. Future studies should examine these additional proteins and other potential pathways to reveal further mechanisms underlying the ALL-BM-MSC interactions.

## Conclusion

Our results indicate that the bone marrow microenvironment changes following cell-cell contact, which subsequently increases the survival of leukemic cells. Additionally, interaction with leukemia cells activates the Wnt/β-catenin pathway in MSCs, with increased nuclear β-catenin expression suggesting a series of changes in gene expression within the cell, potentially influencing leukemia progression. In this context, beta-catenin may play a targetable role in complex cell-cell interactions between ALL blasts and MSCs, presenting an attractive target for novel drug therapies.

## Data Availability

The data that support the findings of this study are available from the corresponding author upon request.
